# The Era of Preemptive Medicine: Developing Medical Digital Twins through Omics, IoT, and AI Integration

**DOI:** 10.31662/jmaj.2024-0213

**Published:** 2024-11-11

**Authors:** Tadao Ooka

**Affiliations:** 1 Department of Health Sciences, University of Yamanashi, Chuo, Japan; 2 Department of Emergency Medicine, Massachusetts General Hospital, Harvard Medical School, Boston, USA

**Keywords:** Artificial intelligence, Deep learning, Digital twin, Internet of Things, Machine learning, Multi-omics, Personalized medicine, Preventive medicine

## Abstract

Preemptive medicine represents a paradigm shift from reactive treatment to proactive disease prevention. The integration of omics technologies, the Internet of Things (IoT), and artificial intelligence (AI) has facilitated the development of personalized, predictive, and preemptive healthcare strategies. Omic technologies, such as genomics, proteomics, and metabolomics, provide comprehensive insights into molecular profile of an individual, revealing potential disease predispositions and health trajectories. IoT devices, such as wearables and smartphones, enable continuous and periodic monitoring of physiological parameters, thus providing a dynamic view of an individual’s health status. AI algorithms analyze comprehensive and complex data from omics and IoT technologies to identify patterns and correlations that inform predictive models of disease risk, progression, and response to interventions. Medical digital twins, or virtual replicas of an individual’s biological processes, have emerged as the cornerstone of preemptive medicine. The integration of omics, IoT, and AI enables the development of medical digital twins, which in turn allows for precise simulation of human physiological profiles, prediction of future health outcomes, and virtual individual clinical trials, facilitating personalized proactive interventions and preemptive disease control. This review demonstrates the convergence of omics, IoT, and AI in preemptive medicine, highlighting their potential to revolutionize healthcare by enabling early disease detection, personalized treatment strategies, and chronic disease prevention. We show how AI leverages omics and IoT in preemptive medicine through several case studies while also discussing the necessary data for developing medical digital twins and addressing ethical and social aspects that warrant consideration. Medical digital twins signify a fundamental transformation in health management, shifting from treating diseases after their occurrence to controlling them before their occurrence. This approach enhances the effectiveness of medical interventions and improves overall health outcomes, preparing for a healthier future.

## 1. Introduction

The advent of preemptive medicine marked a major shift in healthcare, with the focus on controlling diseases before they develop rather than treating them after they occur ^(1)^. Traditional medical approaches are reactive, and they address illnesses only after symptoms appear. Moreover, preemptive medicine identifies and mitigate health risks early through advanced technologies and personalized strategies ^(2)^. This proactive approach is gaining momentum with the integration of omics data, the Internet of Things (IoT), and artificial intelligence (AI), which provide unprecedented insights into individual health profiles and potential disease trajectories ^(3)^.

Recent advancements in genomics, proteomics, metabolomics, and other omics fields have greatly expanded our understanding of the biological mechanisms underlying health and disease ^(4)^. These technologies enable the comprehensive analysis of various molecular layers, revealing genetic predispositions and early disease markers. By integrating diverse data types, including genetic sequences and metabolic profiles, omics data provide a comprehensive view of an individual’s health status and potential future health risks ^(5)^. However, continual monitoring of these molecular layers presents challenges for practical applications.

IoT technologies complement these efforts by continuously monitoring health metrics through interconnected devices such as wearable sensors, smartphones, and environmental monitoring devices ^(6), (7)^. These devices collect and transmit data on vital signs, physical activity, environmental conditions, and healthcare information, providing a dynamic and continuous picture of an individual’s health. Integrating IoT and omics data enhances the precision of health monitoring and enables timely interventions based on real-time metrics ^(8)^.

AI, which has predictive, pattern recognition, and data imputation capabilities, effectively addresses the challenges of volume, complexity, and sparsity of omics and IoT data ^(9)^. The proposed method identifies patterns and correlations in large medical datasets, providing insights and discoveries beyond human analytical capabilities. Applying AI to integrate omics and IoT data allows researchers to develop predictive models to predict disease risk, progression, and intervention response ^(10), (11)^. These models enhance the precision of predictive analytics and enable personalized healthcare strategies.

In addition, the concept of medical digital twins, or health digital twins, is emerging as a powerful tool for personalized and precision medicine ^(12), (13)^. By integrating omics, IoT data, and AI, medical digital twins can create a digital replica of an individual’s biological processes, enabling continuous monitoring and simulation of health scenarios ^(14)^. This approach transforms healthcare from focusing on diseases curing to preemptively simulating and regulating disease states.

This study demonstrated the importance of integrating omics, IoT, and AI technologies into preemptive medicine. This begins by defining preemptive medicine and discussing its historical development and current state. Then, it delves into the various types of omics data, highlighting their characteristics and the importance of integration. The role of IoT in health data collection and use was examined, emphasizing how continuous health monitoring can enhance preemptive healthcare strategies. The role of AI in analyzing these complex datasets and developing simulation models is discussed and supported by case studies that demonstrate successful applications. Strategies for integrating omics, IoT, and AI data to develop digital medical twins are explored, along with challenges and potential solutions in this rapidly evolving field. Finally, the ethical and social implications of such technologies and their potential to reshape the future of healthcare are considered.

## 2. Concept of Preemptive Medicine

Preemptive or proactive medicine predicts and regulate diseases before they fully develop ^(15), (16)^. This approach is based on the understanding that early intervention can significantly improve health outcomes and reduce the overall healthcare system burden. By identifying risk factors and early disease markers, healthcare providers can implement strategies to prevent or delay disease onset, thereby enhancing quality of life ^(17)^.

The concept of preemptive medicine, rooted in historical practices of early disease detection and intervention ^(18)^, has significantly advanced with the advent of genomic and other omics technologies ^(19)^. The completion of the Human Genome Project in 2003 was pivotal because it provided a comprehensive map of the human genome and preparing for personalized medicine ^(20)^. This breakthrough has enabled researchers to identify genetic predispositions to various diseases and understand their mechanisms at the molecular level ^(21)^.

Currently, preemptive medicine incorporates a wide array of data sources, such as genomic, proteomic, metabolomic, transcriptomic, epigenomic, and microbiomic data, to provide a holistic view of an individual’s health ^(22), (23), (24), (25)^. This multi-omics approach provides a deeper understanding of the interactions between different biological systems and how they contribute to disease. By analyzing these diverse datasets, healthcare providers can identify early disease manifestations and underlying pathobiology, thereby tailoring interventions to each individual’s unique biological profile.

IoT technologies are integral to preemptive medicine, enabling continuous monitoring of health metrics through interconnected devices such as wearable sensors, smartphones, and environmental monitoring devices ^(6), (26)^. These devices and technologies collect and transmit data on vital signs, physical activity, sleep patterns, environmental conditions, and other health indicators―information that cannot be captured in hospitals or health checkup facilities―to provide a dynamic and continuous picture of an individual’s health status. Integrating IoT and omics data enhances the precision of health monitoring and enables timely preventive interventions based on continuous health metrics ^(27), (28)^.

Despite the promising potential of preemptive medicine, a few challenges remain. Integrating and interpreting vast numbers of omics and IoT data is complex and requires sophisticated analytical tools and expertise ^(29)^. Another major challenge is the lack of established systems for longitudinally collecting and integrating these diverse datasets, on which AI and machine learning algorithms rely ^(30)^. In addition, ethical and social implications, such as data privacy, informed consent, and equitable access, must be addressed to ensure that preemptive medicine benefits are realized responsibly ^(31), (32)^.

The construction of medical digital twins by integrating multi-omics, IoT data, and AI is a powerful driver for the rapid implementation of preemptive medicine, enabling the precise simulation of individual health conditions and preemptive regulation of disease states before their onset ^(12), (13), (14), (33)^. Although various initiatives are underway, continued technological advancements and interdisciplinary collaboration will be vital to overcome current challenges and realize the full potential of preemptive medicine.

## 3. Omics Data in Preemptive Medicine

Omics data comprise various types of biological information and provide comprehensive insights into molecular components and their interactions within organisms. The integration of omics data has revolutionized preemptive medical strategies, offering unprecedented opportunities for early disease detection and personalized intervention ^(23)^.

**Genomics** is the study of an organism’s complete DNA set. It is crucial to identify genetic predispositions to diseases. High-throughput sequencing technologies, such as whole-genome and exome sequencing, enable rapid analysis of genetic variations. Recent studies have demonstrated that whole-genome sequencing can identify pathogenic variants in many presumably healthy individuals, supporting its potential for personalized risk assessment and targeted prevention strategies ^(34)^.

**Transcriptomics** is the study of RNA transcripts. It helps researches understand gene expression patterns associated with health and disease states. Recent applications of RNA sequencing have identified distinct cell populations and gene expression signatures in various diseases, providing insights into disease heterogeneity and identifying potential targets for early intervention ^(35)^.

**Proteomics** focuses on large-scale proteins studies and provides insights into cellular functions and disease mechanisms. Advanced techniques such as mass spectrometry facilitate the identification of protein biomarkers. Previous studies have used high-throughput proteomics to identify proteins that can predict the risk of cardiovascular events more accurately than traditional risk factors, highlighting the potential of proteomic biomarkers for early disease detection and risk stratification ^(36)^.

**Metabolomics** examines small molecules involved in metabolism and provides real-time snapshots of their biochemical activities. Studies have shown that metabolomic profiling can identify early markers of insulin resistance and type 2 diabetes, such as branched chain and aromatic amino acids, years before disease onset ^(37)^. This approach demonstrates the potential of metabolomics to reveal early signs of metabolic disorders and support preemptive interventions.

**Epigenomics** investigates chemical modifications of DNA and histones that regulate gene expression without altering the DNA sequence. Recent advances in epigenomic profiling technologies have revealed the complex patterns of epigenetic alterations associated with various diseases ^(38)^. For example, integrative analysis of DNA methylation, histone modification, and chromatin accessibility has demonstrated its ability to predict the risk and progression of multiple chronic diseases, such as cardiovascular disorders and certain cancers ^(38)^.

**Microbiomics** examines the collective genomes of microorganisms in specific environments, such as the gut, oral, and skin microbiota. Multi-omics studies of inflammatory bowel diseases, such as Crohn’s disease and ulcerative colitis, have revealed significant heterogeneity and identified key microbial, biochemical, and host factors involved in disease activity ^(39)^. The results of this study highlight the potential of microbiome analysis for developing diagnostic and therapeutic approaches for complex diseases.

**Figure 1.**
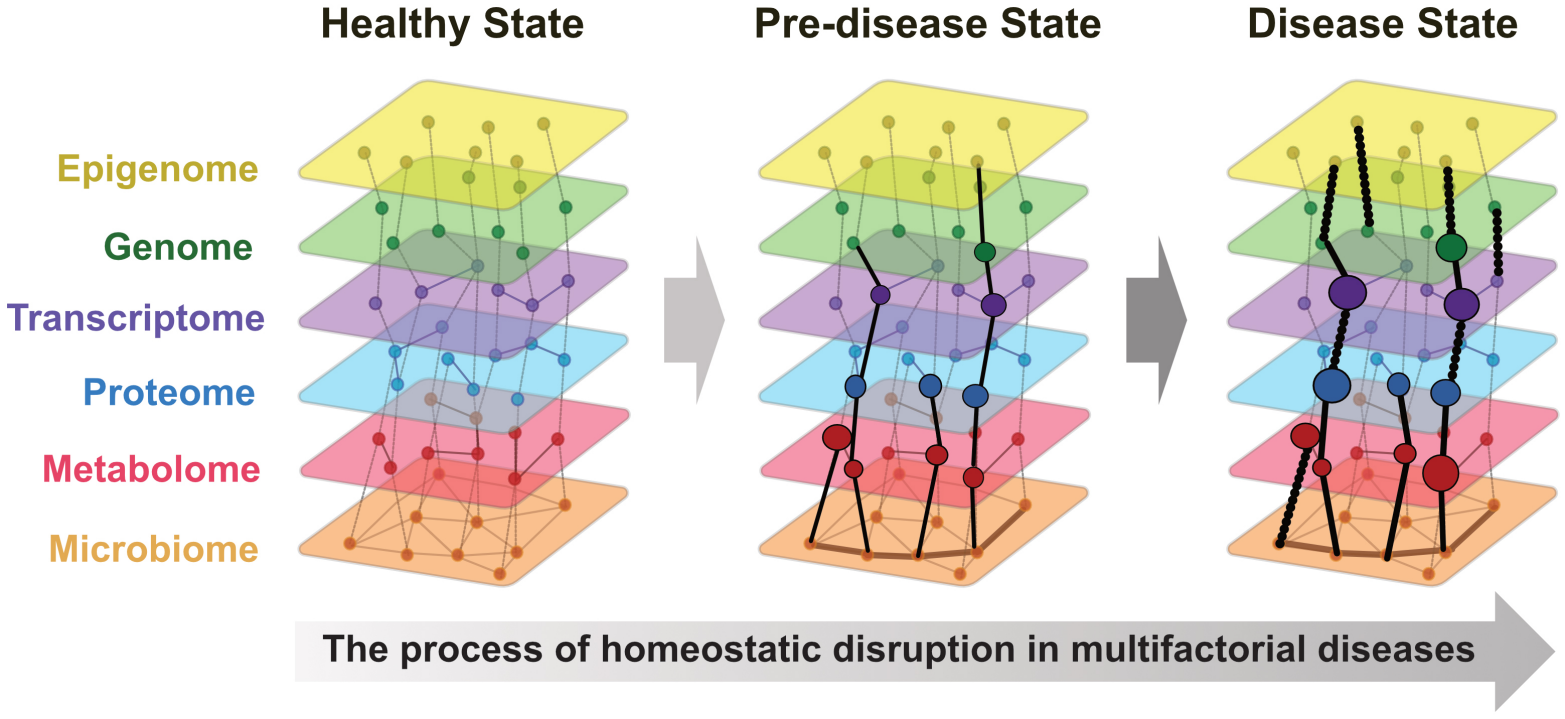
Multi-omics representation of homeostatic disruption in multifactorial diseases The stages of homeostatic disruption in multifactorial diseases have been shown through the integration of multi-omics data. Multi-omics layers show the transition from a healthy state, characterized by stable and balanced omics layers (epigenome, genome, transcriptome, proteome, metabolome, and microbiome), to a predisease state, where slight disruptions and interactions between layers appear, and finally to a disease state, marked by significant disruptions and altered interactions. This process emphasizes how multi-omics data can represent the continuum from health to disease onset.

Integrating these diverse omics data, known as multi-omics, offers a holistic approach to understanding biological systems. A recent study suggested that many conditions, particularly multifactorial diseases, arise from breakdown of homeostasis ^(40)^. Multi-omics data allows continuous representation from a healthy state through predisease to disease onset (Figure 1). In addition, a comprehensive review highlighted advancements in multi-omics data integration techniques, such as deep learning and network-based approaches ^(41)^. This study demonstrated the successful application of these advanced computational methods to unravel the complex disease mechanisms of cancer, neurodegenerative disorders, and metabolic diseases. These findings emphasize the importance of integrating multi-omics and AI to advance precision and preemptive medicine.

## 4. IoT-based Preemptive Medicine

The IoT is considered a transformative technology in healthcare, enabling the continuous collection and monitoring of health metrics. This network of interconnected devices, such as wearable sensors, smartphones, and environmental monitoring devices, provides valuable insights into an individual’s physiological state and lifestyle, significantly contributing to the implementation of preemptive medicine ^(6)^.

Wearable devices, such as fitness trackers and smartwatches, are the most common IoT technologies for preemptive medicine. They monitor vital signs such as heart rate, blood pressure, and physical activity levels. For example, devices such as the Apple Watch and Fitbit can track physical activity and sleep patterns and have advanced features for detecting irregular heartbeats and other anomalies ^(42)^. Similarly, continuous glucose monitoring is used by patients with diabetes to track their blood sugar levels in real time, providing critical data for preemptively managing their condition ^(43)^.

Intermittent data collected by IoT devices are also invaluable in preemptive medicine. Periodic monitoring enables the early detection of abnormal patterns that may indicate the onset of a health issue. For instance, a consistent increase in the resting heart rate could indicate the onset of cardiovascular problems, prompting early intervention^(44)^. In addition, IoT devices that can monitor medication adherence should ensure that patients take their medications as prescribed, which is crucial for preventing the exacerbation of chronic diseases ^(45)^.

Integrating IoT data with electronic health records (EHRs) and other health record systems enhances the comprehensiveness of patient data, enabling healthcare providers to make informed decisions about preemptive care. AI algorithms can analyze IoT data to identify trends and correlations that may not be obvious using traditional data analysis methods, thereby facilitating personalized health recommendations and tailored preventive strategies ^(46)^.

Several successful implementations of IoT in preemptive healthcare have demonstrated its potential, particularly in managing specific chronic conditions ^(47), (48)^. For example, remote patient monitoring systems have significantly improved the management and prevention of disease exacerbations. A recent systematic review showed that IoT-based interventions, particularly remote monitoring, can significantly reduce hospital admissions and improve the quality of life of patients with chronic conditions, such as heart failure and chronic obstructive pulmonary disease (COPD) ^(47)^. The COVID-19 pandemic has accelerated the adoption of IoT technologies in healthcare, highlighting their importance in maintaining continuity of care while minimizing in-person contact. Telehealth solutions integrated with IoT devices have become increasingly prevalent, allowing remote monitoring and virtual consultation ^(48)^.

Despite these benefits, adopting IoT in preemptive healthcare faces several challenges, particularly with data privacy and security as data collection and sharing increases. Although recent regulations and guidelines have been developed to address these issues, there are still problems with implementation and compliance ^(49)^. However, the future of the IoT in preemptive medicine is promising. Advances in 5G technology, edge computing^*^, and AI have enhanced the capabilities of IoT devices, making them more accurate, responsive, and user-friendly for preventive care. Integrating IoT with other emerging technologies, such as blockchain for improved data security and virtual reality for rehabilitation, shows potential for further revolutionizing preemptive healthcare practices ^(50)^.

^*^Edge computing means processing data close to where it is created, such as on IoT devices, instead of sending it to a distant cloud server. This helps reduce delays and the amount of data that must be sent, thereby making the system faster and more efficient. This is especially useful for tasks such as directly simulating health conditions on individual devices.

## 5. The Role of AI and the Concept of Medical Digital Twins

AI continues to revolutionize preemptive medicine by enhancing the analysis and interpretation of vast amounts of data from omics technologies and IoT devices. Recent advancements in machine learning and deep learning algorithms have significantly improved our ability to identify complex patterns and correlations in large datasets, enabling more accurate prediction of disease risk and progression ^(51)^.

### 5-1. The role of AI in omics research

AI technologies have been widely applied to the analysis of omics data for disease onset prediction, prognosis estimation, disease clustering, and identification of pathobiological profiles. These advancements have led to preventive strategies that identify groups at high risk of disease development and enable preemptive, tailored interventions for each group. AI models have demonstrated remarkable ability to extract meaningful patterns from high-dimensional omics datasets in various fields ^(52), (53), (54), (55), (56), (57)^.

AI models have been developed for each omics layer, particularly genomics, proteomics, and metabolomics, to predict disease risks and enable proactive health management. A recent study introduced AD-DL PRS, a deep learning-based polygenic risk score model for predicting Alzheimer’s disease, which outperformed traditional polygenic risk scores in predicting disease onset ^(52)^. Advanced machine learning techniques enhance biomarker discovery, pathway analysis, and endotype clustering in proteomics. Studies have improved the interpretability and identification of key proteins and pathways in complex conditions, such as sepsis and COVID-19 ^(53)^, and have used unsupervised learning to analyze the proteomic profiles of infants with severe bronchiolitis, identifying distinct endotypes with differential risks of childhood asthma development ^(54)^. A pervious study used machine learning algorithms to develop metabolomic panels for gastric cancer diagnosis and prognosis, enabling early detection with sensitivity surpassing that of conventional methods and providing guidance for precision interventions through patient risk stratification ^(55)^.

The impact of AI on preemptive medicine is further amplified when applied to integrated omics analysis, leading to more comprehensive and personalized preventive strategies. For instance, a study demonstrated the potential of integrated omics analysis in respiratory syncytial virus bronchiolitis by combining clinical, viral, airway microbiome, transcriptome, and metabolome data to identify biologically distinct endotypes with differential risks of childhood asthma development ^(56)^. In addition, network-based approaches to multi-omics data integration provide a more intuitive representation of complex biological relationships and promising directions for field development. For instance, MOGONET (multi-omics graph convolutional networks) leverages the power of graph convolutional networks in conjunction with deep learning techniques to integrate multi-omics data, demonstrating superior performance in biomedical classification tasks ^(57)^. These integrated approaches enhance the understanding of disease mechanisms and enable early identification of individuals at high risk. They transform preventive medicine into preemptive medicine by facilitating earlier, more precise risk assessment and personalized prevention strategies for various conditions.

### 5-2. The role of AI in IoT research

Integrating AI with IoT technologies has opened new frontiers in preemptive medicine, enabling continuous health monitoring, early disease detection, and personalized interventions. AI algorithms, particularly machine learning and deep learning models, effectively extract meaningful patterns from vast amounts of data generated by IoT devices, leading to more accurate predictions and timely interventions ^(58), (59)^.

In cardiovascular health, AI-powered IoT systems show promise for the early detection and prevention of cardiac events. A recent study developed a deep learning model called warning of atrial fibrillation, which predicts atrial fibrillation (AF) at an average of 30.8 min before onset with an accuracy and F1 score of 83% and 85%, respectively. This model, which uses R-to-R interval signals from wearable devices, demonstrates the potential of early AF detection ^(60)^. Another study evaluated an AI-enabled ECG alert system in a randomized trial involving 39 physicians and 15,965 patients. The system, providing AI reports and warnings for patients at high risk, significantly reduced 90-day all-cause mortality (3.6% vs. 4.3% in the control group), especially in patients at high risk ^(61)^.

AI-IoT systems are instrumental in chronic disease management by enabling proactive monitoring and prediction of exacerbations. An AI-powered remote monitoring system for patients with COPD is feasible for facilitating the early detection of exacerbations and timely intervention to prevent hospitalization ^(62)^. In diabetes care, an AI-driven closed-loop system that simulates an artificial pancreas has shown the potential to revolutionize management by autonomously regulating blood glucose levels ^(63)^.

The potential of AI-IoT integration extends beyond individual health monitoring to population-level disease surveillance and outbreak prediction. A pervious study demonstrated how AI models can analyze data from IoT devices and social media to predict influenza outbreaks more accurately and lead times than traditional surveillance methods ^(64)^. This approach exhibits the potential of AI-IoT systems to enable proactive public health measures and resource allocation.

### 5-3. Concept and development of medical digital twins

Medical digital twins or health digital twins represent an emerging frontier in personalized and preemptive medicine. They are comprehensive virtual replicas of an individual’s biological system, integrating various longitudinal data sources to create a dynamic model of the patient’s health ^(13), (14)^. This technology holds great promise for improving patient-specific treatments and diagnostics by enabling more accurate phenotyping or endotyping of patients with similar presentations or conditions. Recent advancements in big data, data science, and AI have been crucial in making medical digital twins a reality, providing the necessary infrastructure for processing and analyzing the vast amounts of data required to create and maintain these complex models ^(65)^.

**Figure 2.**
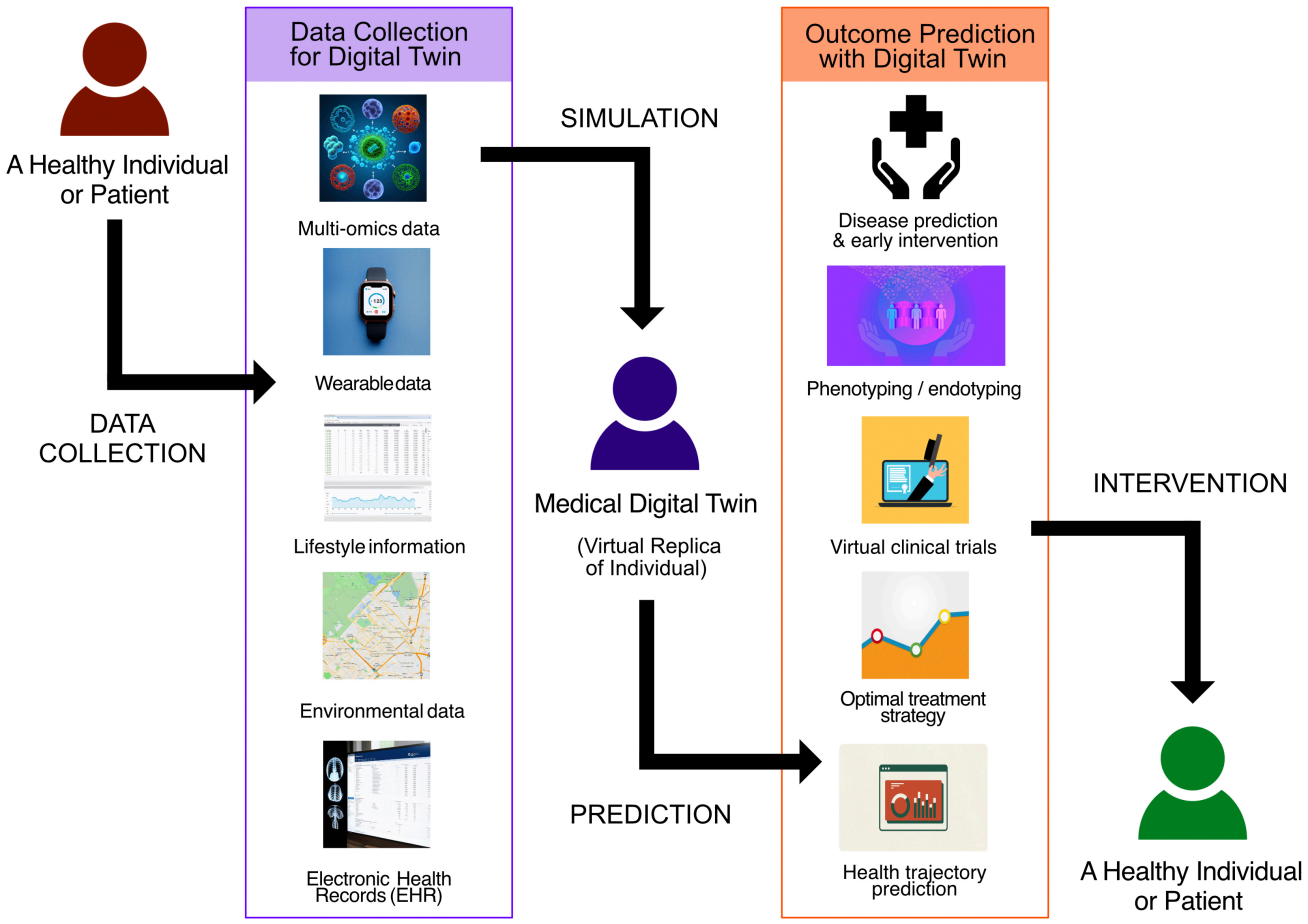
Creation and application of a medical digital twin Data such as multi-omics data, wearable data, lifestyle information, environmental data, and electronic health records are collected from a healthy individual or patient. These data are used to create a medical digital twin, which serves as a virtual replica of the individual and enables various outcome predictions. The predictions include disease prediction and early intervention, phenotyping/endotyping, virtual clinical trials, optimal treatment strategies, and health trajectory prediction. The resulting insights can guide personalized interventions for the individual.

The development of medical digital twins builds on advancements in omics technologies and IoT devices. The twins were constructed by combining longitudinal data from multiple sources (Figure 2).

1. Multi-omics data: genomics, epigenomics, transcriptomics, proteomics, metabolomics, microbiomics, and other molecular-level information

2. Wearable data: continuous health monitoring of wearable devices and smart sensors

3. Lifestyle information: diet, exercise, smoking habits, alcohol consumption, bowel movements, and other lifestyle behaviors

4. Environmental data: geographic positioning system (GPS)-based or geographic information system (GIS)-based information about air quality, temperature, humidity, and other environmental factors

5. EHR: clinical history, diagnoses, treatments, outcomes, and medical images (such as X-ray images, CT scans, MRI scans, and pathology images)

In this study, IoT data refer to data collected from IoT devices, including items 2-4. By integrating these diverse data types, medical digital twins can provide a comprehensive real-time representation of an individual’s health status in the environment ^(14), (66)^.

The potential applications of medical digital twins in healthcare are extensive and evolving. In cardiovascular medicine, these models can predict conditions and optimize treatment selection for individual patients by constructing a virtual representation of a patient who receives real-time updates from various data sources ^(67)^. Similarly, in diabetes management, digital twins offer a promising approach to address the challenge of limited healthcare resources, potentially improving personalized treatment strategies and optimizing resource allocation ^(68)^. In drug development and personalized medicine, digital twins can be treated computationally with thousands of drugs, accelerating the process of finding optimal treatments for individual patients across a wide range of conditions ^(12)^.

As the field progresses, ongoing research has focused on improving the accuracy and comprehensiveness of such models. The future of healthcare may involve more advanced methods for optimal patient treatment, with digital twins playing a crucial role in planning during the post-digital era ^(69), (70)^. Although still in early development, digital medical twins represent a promising approach for achieving precision and preemptive medicine, potentially significantly improving healthcare outcomes and patient quality of life by leveraging the power of multi-omics, IoT data, and AI.

## 6. Social Implementation of Preemptive Medicine

The integration of multi-omics, IoT, and AI has paved the way for a paradigm shift in healthcare toward preemptive medicine. This approach transitions from reactive to proactive healthcare, significantly improve patient outcomes, and reduce healthcare systems burden. Global initiatives are advancing this transition, bringing us closer to the future of personalized, predictive healthcare.

### 6-1. Current initiatives and projects

In the United States, the All of Us Research Program, which is part of the Precision Medicine Initiative, aims to create a cohort of one million volunteers who will contribute their health data and biospecimens to a centralized national database. This ambitious project supports precision medicine research and addresses the crucial ethical, legal, and social issues associated with large-scale data collection ^(71)^.

Singapore’s PRECISE-SG100K study is another comprehensive, long-term initiative focusing on the health and well-being of a diverse, multiethnic population. By collecting detailed health information and biological specimens from more than 100,000 participants, this project aimed to identify the social, environmental, lifestyle, and genetic factors associated with various diseases, providing insights into their local and global significance ^(72)^.

In Japan, two notable projects have made significant strides toward preemptive medicine. The NTT Research MEI Lab is dedicated to advancing personalized, preventive, predictive, and participatory (P4) medicine ^(1)^ by developing bio-digital twin technologies. Their initial focus was precision cardiology, with plans to expand to wellness and prevention ^(73)^. Concurrently, the Yamanashi Multi-omics Cohort (YMoC) study combined longitudinal multi-omics data with real time and periodic health metrics collected from wearable devices and a smartphone application to identify early biomarkers of chronic diseases. Their innovative approach represented medical digital twins on individual smartphones, enabling AI-driven personalized behavioral interventions ^(74)^. These initiatives demonstrate the global effort to realize preemptive medicine by integrating various technologies and data types, each contributing uniquely to the advancement of personalized, predictive healthcare.

### 6-2. Ethical and social aspects

As we move toward the implementation of preemptive medicine, it is crucial to address the ethical and social aspects of integrating advanced technologies. Given the sensitive nature of health data, it is very important to ensure data privacy and security ^(71)^. Robust encryption methods, access controls, and anonymization techniques are essential to protect patient information. In addition, regulatory frameworks must evolve to address these challenges and ensure the ethical use of medical digital twins ^(32), (75)^.

Implementing preemptive medicine hinges on maintaining robust privacy protection while facilitating researchers’ access to vast amounts of sensitive data, such as DNA analysis results, EHRs, behavioral assessments, and mHealth data, which capture “digital traces” of participants’ daily lives ^(32)^. A state-of-the-art IT security system with robust institutional policies is essential; however, challenges persist due to the unprecedented scale and diversity of the data collected. Novel issues arise from mHealth data, which blur the lines between health and nonhealth information. The key ethical, legal, and social implications of this approach include the potential misuse of data by unauthorized parties, the need for firewalls between the research program and other government agencies to prevent inappropriate data sharing, unanticipated revelations from combined data sources, and the integration of research findings into clinical care ^(71)^.

A recent study ^(75)^ identified four key ethical and social concerns in implementing digital twins for personalized healthcare services: (1) data collection issues, such as overcollection, privacy infringement, and potential coercion in monitoring patient adherence; (2) data management challenges, such as accessibility, ownership, and risks associated with data brokerage and cybersecurity; (3) data analysis concerns, such as algorithmic bias and discrimination potential, especially for underrepresented populations; and (4) information use problems, such as the decontextualization of disease formation, epistemic injustice, and risks of overdiagnosis and overtreatment. These challenges span various phases of development and use, underscoring the need for careful consideration and mitigation strategies to ensure that digital twin technologies empower patients and improve their health outcomes without compromising individual rights or exacerbating existing healthcare disparities.

Public perception and acceptance are vital in successfully implementing preemptive medical technologies. It is necessary to educate the public on the benefits and limitations of multi-omics, IoT data, and AI integration in healthcare in order to build trust and acceptance ^(13), (14)^. Addressing concerns on data privacy, security, and the ethical use of these technologies will help alleviate fear and promote more informed public discourse. As we progress toward realizing medical digital twins and preemptive medicine, interdisciplinary collaborations among biologists, data scientists, engineers, healthcare professionals, and ethicists will be essential ^(32), (75)^. These collaborations drive technological advancements while ensuring that ethical and social considerations are proactively addressed.

## 7. Conclusion

The integration of multi-omics, IoT data, and AI is transforming preventive medicine from reactive to proactive strategies. These technologies enable the development of digital medical twins, offering unprecedented opportunities for personalized interventions. However, they have significant ethical and social considerations. Addressing these challenges requires translational collaboration among diverse experts to improve these efforts responsibly.

We can reduce the disease burden, minimize invasive treatments, and enhance the overall quality of life by leveraging advanced technologies for prediction and prevention. Continued investment in research, development, and ethical implementation will prepare for personalized, predictive, and preemptive healthcare, ultimately transforming the human experience of health and well-being.

## Article Information

### Conflicts of Interest

None

### Acknowledgement

This manuscript was developed with the support of AI-assisted tools, such as ChatGPT-4o (OpenAI, Inc., CA, USA), Gemini Advanced (Google LLC, CA, USA), and Claude 3.5 Sonet (Anthropic, CA, USA), which facilitated grammar correction, refinement of expression, and overall improvement in the quality of the manuscript. The figures in this manuscript were primarily created using Affinity Designer 2 (Serif Europe Ltd, Nottingham, UK), with the elemental images in Figure 2 specifically generated using the AI-assisted tool, Stable Diffusion 3 Medium (Stability AI Ltd, London, UK).
